# Awareness and Practices Related to Cervical Cancer among Females in Saudi Arabia

**DOI:** 10.3390/ijerph19031455

**Published:** 2022-01-27

**Authors:** Heba M. Zahid, Alma B. Qarah, Amal M. Alharbi, Arwa E. Alomar, Shaimaa A. Almubarak

**Affiliations:** Department of Medical Laboratory Technology, College of Applied Medical Sciences, Taibah University, Al Madinah Al Munawwarah 42353, Saudi Arabia; alma.qarah@gmail.com (A.B.Q.); Amal.bshibsh@gmail.com (A.M.A.); arwa.essam08@hotmail.com (A.E.A.); shaimaa1abdullah@gmail.com (S.A.A.)

**Keywords:** cervical cancer, knowledge, Al Madinah Province, Saudi Arabia

## Abstract

Human papilloma virus (HPV) is the most common risk factor for cervical cancer. Cervical cancer can be prevented with vaccination and early screening methods using pap smears. However, the acceptance of these approaches can be affected by the awareness level of the population. This cross-sectional study aimed to assess knowledge and practices related to cervical cancer among women in the Al Madinah Province in Saudi Arabia. A total of 1489 responses were included in the analysis. The median awareness score related to cervical cancer was eight out of 20 points. Vaginal bleeding, dyspareunia, and leg pain were correctly identified by 79.8%, 43.7%, and 19.3% of the women, respectively. Thirty-four percent of the study sample knew that the sexually transmitted virus is a risk factor for cervical cancer. Only 44.6% were able to correctly identify pap smear as a screening tool, and 12.6% knew that there was a HPV vaccine. This study revealed a low to moderate awareness level toward cervical cancer, pap smear, and HPV vaccine. Thus, awareness campaigns are urgently needed to increase the awareness level for early detection and prevention of the disease.

## 1. Introduction

Cervical cancer is the fourth most common female cancer worldwide and the ninth most common in Saudi Arabia [1,2]. One of the most common risk factors for developing cervical cancer is human papilloma virus (HPV), more specifically, HPV-16 and HPV-18. Other risk factors include smoking, immunodeficiency, chlamydia infections, long-term use of birth control pills, multiple pregnancies, and low socioeconomic status [3]. Cervical cancer is asymptomatic at early stages, yet it is highly curable [4]. Thus, early diagnosis is critical for better prognosis. The Papanicolaou (Pap) smear screening method identifies any cytological abnormalities in the cervix. Therefore, the pap smear is a lifesaving procedure as early detection leads to better outcomes.

Although trends in cervical cancer incidence have shown a steady decline globally, the incidence has increased in Saudi Arabia by 453.6% since 1990 with 358 new cases yearly and 179 deaths [5,6]. Effective screening and vaccination programs have affected the global trend. For instance, after the introduction of the HPV immunization program in England, a massive reduction in cervical cancer cases was observed [7]. Additionally, after the implementation of an organized cervical cancer screening program in Europe, a 51–92% reduction was observed in cervical cancer mortality in different parts of Europe [8]. However, such a result depends mainly on the awareness level of the population and their acceptance of performing the screening test or receiving the vaccine. This was reported in a systematic review where cervical cancer educational intervention significantly improved cervical cancer screening rates [2]. Thus, multiple studies have highlighted the limited awareness regarding cervical cancer screening and vaccination in different populations [9,10,11,12,13,14].

In Saudi Arabia, cervical cancer screening is recommended after marriage when women become sexually active. However, there is no comprehensive screening program, and it depends on the preference of the woman and the recommendations of her healthcare provider. As a result, 43% of the cervical cancer cases in Saudi Arabia are diagnosed at stage III and IV compared to 25% in British Colombia where they have an effective screening program [15]. Regarding HPV immunization in Saudi Arabia, it is available from the age of 9 to 26 years. However, it was only added recently to the immunization records of girls and boys from 9–12 years old, which will eliminate cervical and penile cancer cases in the future.

Data regarding awareness level were measured in university students and the general population in Riyadh, Jeddah, Al-Ahsa, Hail, Qassim, and the southern region [16,17,18,19,20,21,22,23,24]. However, different results were obtained, suggesting that the knowledge level varies among women as a function of their occupation or region. Therefore, the current study aimed to assess knowledge and practices related to cervical cancer among women in the Al Medina Al Munawara region in Saudi Arabia. Findings of this study will assist in the development of customized awareness campaigns, as well as screening and vaccination programs, in order to limit the incidence of cervical cancer among women in Saudi Arabia.

## 2. Materials and Methods

### 2.1. Study Design and Population

A cross-sectional study was used and women 18 years or older who currently reside in Al Madinah Province in Saudi Arabia were recruited. The sample size required to obtain a 95% confidence interval with a 3% margin of error was 1066 women. A total of 1644 responses were received. However, a total of 12 responses were excluded (0.80%) for incomplete data and 134 responses (8.95%) were excluded for unmatched inclusion criteria (e.g., male, non-Saudi, and <18 years). The remaining 1498 responses were included in the analysis. Consent of participation in this study was obtained from each participant online before data were collected. Ethical approval was obtained from the College of Applied Medical Sciences Ethical Committee at Taibah University, Medina, Saudi Arabia (SREC/AMS 2019/45/CLD).

### 2.2. Data Collection

Data were collected using an online self-reported questionnaire. The questionnaire was provided in Arabic, and it was distributed through multiple social media applications (WhatsApp and Twitter). Family, friends, staff, and students of Taibah University from different campuses were contacted to help in the survey distribution. In addition, participants from disadvantaged communities were also targeted to ensure a fair representation of the study population.

### 2.3. Data Collection Instrument

#### 2.3.1. Sociodemographic Characteristics

The online questionnaire collected data concerning sociodemographic characteristics, including age group (18–25 years = 1, >25 years = 2), marital status (single = 1, married, divorced, or widowed = 2), education level (≤high-school/diploma = 1, bachelors/postgraduate degree = 2), employment status (employee or student in medical field = 1; employee or student in nonmedical field = 2, not employee or student = 3), family income per month (<10,000 SAR = 1, 10,000 SAR = 2, >10,000 SAR = 3), and region of residency (Al Madinah Al Munawwarah city = 1, other cites = 2).

#### 2.3.2. Awareness Related to Cervical Cancer

The second section of the questionnaire included a total of 20 questions/items to assess the awareness related to cervical cancer. Responses to all items in this section were given a score (depending on response being correct or not correct) in order to transfer responses to the 20 questions into a continuous variable expressing the total awareness level with the total ranging between 0 and 20 points. The 20 items were as follows: symptoms of cervical cancer, with responses of (1) dyspnea, (2) headache, (3) leg pain, (4) pain during urination, (5) dyspareunia, and (6) vaginal bleeding. Participants who correctly selected items 3, 5, and 6 were awarded one point for each item, while participants who did not select these items awarded zero points for each item. Participant who selected wrong items (1, 2, and 4) were awarded zero points, while participants who did not select these items were awarded one point for each item; risk factors of cervical cancer, with responses of (7) family history, (8) virus transmitted sexually, (9) bacteria transmitted sexually, (10) long-term use of contraceptives, (11) multiple birth, and (12) smoking. Participants who correctly selected items 7, 8, 9, 10, 11, and 12 were awarded one point for each item, while participants who did not select these items awarded zero points for each item; additional items to assess the awareness related to pap smear test, such as (13) heard of the pap smear test (yes = 1, no = 0); (14) if yes, what is the pap smear test? (14.1) test to detect cervical cancer; (14.2) test to detect pregnancy; (14.3) I do not know (IDK); participants who selected response 14.1 were awarded one point, while participants who selected response 14.2 and 14.3 were awarded zero points; (15) accuracy of the pap smear test (correct results = 1, incorrect results = 0); (16) frequency of the pap smear test (every month = 0, every year = 0, every 3 years = 1); (17) places to perform the pap smear test (done by the participant = 0, primary care centers = 0, hospitals = 1, IDK = 0); (18) benefits of early screening for cervical cancer (treatment will be easier = 0, signs and symptoms of cancer are not noticeable = 0, cancer prevention = 0, all of the above = 1, IDK = 0); (19) did you hear about the HPV vaccine? (yes = 1, no = 0); (20) what is the appropriate age to receive the HPV vaccine? (≤25 years = 1, >25 years = 0, IDK = 0). An additional item was included in the second section of the questionnaire but was not used to obtain the total awareness score. This item was added to describe data concerning main sources of knowledge related to the HPV vaccine as follows: if you heard about HPV vaccine, what was your main source of information? Responses were “doctor”, awareness campaign”, social media”, and “other sources”.

#### 2.3.3. Practices Related to Cervical Cancer

The last section of the questionnaire included two items to assess practices related to cervical cancer: (1) Have you performed a pap smear test? Responses were “yes” which was coded as 1 and “no” which was coded as 0; (2) Have you received the HPV vaccine? Responses were “yes” which was coded as 1 and “no” which was coded as 0. An additional item was included to provide more descriptive data regarding the reasons for not performing the pap smear test as follows: if you did not perform the pap smear test, what are the reasons for not doing it? Responses were “too shy or uncomfortable to do it”, “do not know where to do it”, “cannot do the test as I have never been married”, “do not know it is important”, and “other reasons for not doing the pap smear test”.

### 2.4. Statistical Analysis

Data are presented as the frequency and percentage (%) for categorical variables and the mean ± standard deviation (SD) and median and interquartile range (IQR) for continuous variables. We assessed the normality of the distribution of total awareness level using the Shapiro–Wilk test. Data of the total score of awareness were found to be skewed. The Mann–Whitney U test was used to compare the mean awareness across the groups (performed the pap smear test vs. did not perform the pap smear test; received the HPV vaccine vs. did not receive the HPV vaccine). Simple linear regression analysis was performed to investigate predictors (e.g., age, family income, education level) of total awareness level related to cervical cancer (outcome). In addition, logistic regression analysis was performed to investigate predictors (e.g., age, family income, education level) of performing the pap smear test (outcome: yes = 1; no = 0). Data analysis presented in this study was performed using the SPSS version 20 (IBM Corp., Armonk, NY, USA). All tests were two-tailed with a confidence level of 99% (α = 0.01).

## 3. Results

### 3.1. Characteristics of Participants

Characteristics of participants included in the study are presented in Table 1. Half of the participants were between the age of 18 and 25 years (*n* = 770), whereas 53.3% (*n* = 799) were married, divorced, or widowed. Two-thirds of the study sample reported having a university or postgraduate degree (67.4%, *n* = 1009). Twenty percent of the study participants were studying or working in the medical field (*n* = 302). Forty-eight percent of participants reported a family income >10,000 Saudi riyal (SAR) (*n* = 722). The majority of participants included in this study were from Al Madinah Al Munawwarah city (80.0%, *n* = 1198).

### 3.2. Awareness and Practices Related to Cervical Cancer

The awareness related to cervical cancer risk factors, testing, and vaccination is described in Table 2. Most participants correctly identified vaginal bleeding as a symptom of cervical cancer (*n* = 1196), while 43.7% and 19.3% correctly identified dyspareunia and leg pain as symptoms of cervical cancer, respectively. Dyspnea, headache, and pain during urination (3.50%, 4.50%, and 29.6%, respectively) were wrongly identified as symptoms of cervical cancer. Participants correctly selected the following as risk factors of cervical cancer: virus transmitted sexually (34.8%, *n* = 522), long-term use of contraceptives (25.0%, *n* = 374), multiple births (4.30%, *n* = 64), smoking (15.0%, *n* = 225), family history (53.5%, *n* = 801), and bacteria transmitted sexually (28.4%, *n* = 426). Over half of the participants had heard about the pap smear test (51.9%, *n* = 778). The majority of participants correctly identified the pap smear test as a test used to detect cervical cancer (85.9%, *n* = 668). Over half of the sample believed that the pap smear test provides incorrect results (55.4%, *n* = 830). Fifteen percent (*n* = 220) indicated that the pap smear test should be done every 3 years. About half of the sample included this study reported that the pap smear test can be done in hospitals (46.5%, *n* = 696). Benefits of early screening for cervical cancer were all identified by 60.9% (*n* = 912) of the sample. Only 12.6% (*n* = 189) had heard about the HPV vaccine, while 9.50% (*n* = 143) correctly identified the appropriate age to receive the HPV vaccine as ≤25 years. Two percent (*n* = 4) reported doctors as their main source of information, while awareness campaigns were the main source of information related to cervical cancer among 8.99% of the study sample (*n* = 17). Mean awareness related to cervical cancer was 8.51 ± 3.06 points and the median was 8.00 points (6.00–11.00) out of 20 points.

Data concerning practices related to cervical cancer show that 12.5% (*n* = 187) of participants performed the pap smear test. Forty-one percent (*n* = 619) of the study sample thought the pap smear test is not important, while 2.70% (*n* = 41) of participants reported they were too shy or uncomfortable to perform the pap smear test. Two-thirds of the sample (*n* = 935) reported that they were willing to pay 300–500 SAR for the pap smear test. Less than 1% (0.20%, *n* = 3) received the HPV vaccine.

### 3.3. Association between Awareness and Practices Related to Cervical Cancer

Mean total awareness was significantly higher among participants who performed the pap smear test previously compared to participants who did not perform the pap smear test (10.1 ± 2.31 vs. 8.29 ± 3.08, respectively, *p* < 0.001). Mean total awareness was significantly higher among participants who had received the HPV vaccine compared to participants who did not receive the HPV vaccine (12.1 ± 2.81 vs. 7.99 ± 2.72, respectively, *p* < 0.001).

### 3.4. Predictors of Awareness and Practices Related to Cervical Cancer

Simple linear regression models were performed for each predictor, and data show that age group (B = 0.43, SE = 0.16 (95% CI: 0.12 to 0.74), *R^2^* = 0.01), marital status (B = 0.55, SE = 0.16 (95% CI: 0.24 to 0.86), *R^2^* = 0.01), employment status (B = −1.21, SE = 0.10 (95% CI: −1.41 to −1.01), *R^2^* = 0.08), family income per month (B = 0.60, SE = 0.09 (95% CI: 0.44 to 0.77), *R^2^* = 0.03), and region of residency (B = −0.55, SE = 0.20 (95% CI: −0.93 to −0.16), *R^2^* = 0.01) predicted the total awareness level related to cervical cancer (Table 3.)

Data show that predictors of performing the pap smear test were age, marital status, employment status, and region of residency (Table 4.). Compared to participants aged 18–25 years, participants aged > 25 years had higher odds of performing the pap smear test (OR = 16.8 (95% CI: 9.66 to 29.3), *p* < 0.001). Compared to single participants, married, divorced, and widowed participants had higher odds of performing the pap smear test (OR = 69.4 (95% CI: 22.1 to 218), *p* < 0.001). Compared to participants with a high school/diploma or lower education, participants with a bachelor’s degree or postgraduate degree had higher odds of performing the pap smear test (OR = 12.0 (95% CI: 0.87 to 1.71), *p* < 0.001). Compared to participants who were employees or students in the medical field, participants who were employees or students in the nonmedical field had higher odds of performing the pap smear test (OR = 2.04 (95% CI: 1.21 to 3.44), *p* = 0.007), as well as participants who were not working or studying (OR = 2.93 (95% CI: 1.75 to 4.92), *p* < 0.001). Compared to participants who were living in Al Madinah Al Munawarah city, participants who were living in other cities in the Al Madinah Province had higher odds of performing the pap smear test (OR = 1.60 (95% CI: 1.13 to 2.27), *p* < 0.001).

Logistic regression analysis could not be performed to investigate predictors of receiving the HPV vaccine as only three participants (0.20%) included in our study took the vaccine.

## 4. Discussion

The availability of an effective early screening method and a successful vaccine has made cervical cancer a preventable disease. To use these benefits and reach zero mortality from cervical cancer, the community knowledge about the screening method and the vaccination should be excellent in order to increase the acceptability of performing the screening or receiving the vaccine. The median awareness score related to cervical cancer in the current study was eight out of 20, equivalent to 40%. This result is close to or lower than what was measured in other Gulf countries such as Oman, Kuwait, and Sharja, with levels of 38.3%, 54%, and 66.2%, respectively [25,26,27]. Countries in Africa showed lower knowledge, as southern and northwest Ethiopia and Cameroon exhibited levels of 26.2%, 30.3%, and 34%, respectively [28,29,30]. On the other hand, some Asian countries had a higher knowledge; for example, levels of 65.7% and 64% were recorded in Vietnam and Indonesia, respectively.

In the current study, 51.9% of the participants had heard about the pap smear. However, only 44.6% (*n* = 668) were able to correctly identify that the pap smear is used for cervical cancer screening, and that it gives accurate results. Similarly, studies from Qassim, the southern region, and Riyadh city showed that 52.5%, 43.5%, and 53.8% of the women had heard about the pap smear, respectively. However, there was no question to confirm if their knowledge was correct [17,23,24]. Another study from Riyadh showed that 79.9% of the women were able to identify that the pap smear is used to detect precancer and cancer of the cervix [31]. The benefits of the pap smear were identified by 61% of the respondents. However, only 14.7% knew that the pap smear should be repeated every 3 years, and 44.3% did not know where they could get a pap smear.

Regarding cervical cancer symptoms, in the current study, vaginal bleeding, dyspareunia, and leg pain were correctly identified by 79.8%, 43.7%, and 19.3% of the women, respectively. Vaginal bleeding and dyspareunia were previously identified by 37.5% and 21.4% of secondary-school teachers in the Al-Hassa region, respectively [32]. However, medical students from the same region had greater knowledge about the signs, as 76.6% and 81.8% of the participants were able to identify vaginal bleeding and dyspareunia as cervical cancer symptoms [22]. Nevertheless, only 54.8% and 65% of medical field students from Riyadh and Hail were able to identify vaginal bleeding as a sign [16,21]. The majority of respondents believed that family history is the main risk factor followed by sexually transmitted virus. In general, all risk factors were identified by the respondents.

It was reported that, in Saudi Arabia, the percentage of women aged 25–49 years who had a pap smear test was 7.6% [33]. However, in the current study, 12.5% of the respondents had done a pap smear. Yet, they might have mistaken it with a vaginal swab as there was no question to validate this. The main reason for not doing a pap smear was not knowing it was important. However, the majority were willing to pay to get the pap smear done. The respondents above 25 years old and married/divorced or widowed had higher odds of performing a pap smear. This could be due to the conservative culture in Saudi Arabia, as women become sexually active only after marriage.

Twelve percent of the respondents had heard about HPV vaccine but only 9.5% were able to identify the recommended age to receive the vaccine. Only three of the respondents had received the vaccine. This is lower than previously reported, as 21% and 33% of female respondents knew that a vaccine for HPV was available in previous studies [14,22]. The Saudi government has taken the initiative to include the HPV vaccine in the immunization schedule for boys and girls aged between 9 and 12 years. This will reduce the cervical cancer cases among the younger generation.

To our knowledge, this is the first study to assess the awareness and practices related to cervical cancer among females in Saudi Arabia. However, the generalizability of the study findings might be limited to females living in the Al Madina Province. Additionally, the convenient sampling technique used to collect data could limit the generalizability of the study findings to literate females who use social media applications regularly.

## 5. Conclusions

Cervical cancer is a preventable disease if vaccination and early screening are applied effectively. The current study revealed low to moderate knowledge about cervical cancer. However, knowledge about vaccination and screening among women of the Al Madina Province was very low. Total awareness was significantly higher in respondents who had performed a pap smear or received the vaccine. A massive awareness campaign should be implemented in schools, universities, healthcare centers and shopping malls to target increasing the knowledge about cervical cancer, specifically, the screening method, its availability, and vaccination. To our knowledge, this is the first study to assess the knowledge and practices related to cervical cancer among women in the Al Madina Province in Saudi Arabia.

## Figures and Tables

**Table 1:** Characteristics of the study participants (*n* = 1498).

	*n*	%
Age		
18–25 years	770	51.4
>25 years	728	48.6
Marital status		
Single	699	46.7
Married/divorced or widowed	799	53.3
Education level		
≤High school/diploma	489	32.6
Bachelor’s degree/postgraduate degree	1009	67.4
Employment status		
Employee or student in medical field	302	20.2
Employee or student in nonmedical field	655	43.7
Not employee or student	541	36.1
Family income per month		
<10,000 SAR	527	35.2
10,000 SAR	249	16.6
>10,000 SAR	722	48.2
Region of residency		
Al Madinah Al Munawwarah city	1198	80.0
Other cites	300	20.0

**Table 2:** Awareness and practices related to cervical cancer (*n* = 1498).

#		*n*	%
Awareness related to cervical symptoms, cancer risk factors, testing, and vaccination			
Symptoms of cervical cancer			
1	Dyspnea	52	3.50
2	Headache	67	4.50
3	Leg pain ^1^	289	19.3
4	Pain during urination	444	29.6
5	Dyspareunia ^1^	654	43.7
6	Vaginal bleeding ^1^	1196	79.8
Risk factors of cervical cancer			
7	Family history ^1^	801	53.5
8	Virus transmitted sexually ^1^	522	34.8
9	Bacteria transmitted sexually ^1^	426	28.4
10	Long-term use of contraceptives ^1^	374	25.0
11	Multiple births ^1^	64	4.30
12	Smoking ^1^	225	15.0
13	Heard of the pap smear test		
Yes ^1^		778	51.9
No		720	48.1
14	If yes, what is the pap smear test?		
Test to detect cervical cancer ^1^		668	85.9
Test to detect pregnancy		19	2.44
I do not know		91	11.7
15	Accuracy of the pap smear test		
Correct results ^1^		668	44.6
Incorrect results		830	55.4
16	Frequency of the pap smear test		
Every 6 months		313	20.9
Every year		317	21.2
Every 3 years ^1^		220	14.7
I do not know		648	43.3
17	Places to perform the pap smear test		
Done by the participant		25	1.70
Primary care centers		114	7.60
Hospitals ^1^		696	46.5
I do not know		663	44.3
18	Benefits of early screening for cervical cancer		
Treatment will be easier		225	15.0
Signs and symptoms of cancer are not noticeable		149	9.90
For cancer prevention		127	8.50
All of the above ^1^		912	60.9
I do not know		85	5.70
19	Heard of the HPV vaccine		
Yes ^1^		189	12.6
No		1309	87.4
If yes, main source of information			
Doctor		4	2.12
Awareness campaign		17	8.99
Social media		64	33.9
Other sources		104	55.0
20	Appropriate age to receive the HPV vaccine		
≤25 years ^1^		143	9.50
>25 years		396	26.4
I do not know		959	64.0
	Practices related to cervical cancer test and vaccine		
1	Done pap smear test		
Yes ^1^		187	12.5
No		1311	87.5
Reasons for not doing Pap smear			
Too shy or uncomfortable to do it		41	2.70
Do not know where to do it		144	9.60
Cannot do the test as I have never been married		285	19.0
Do not know it is important		619	41.3
Other reasons for not doing the test		222	14.8
Amount of money willing to pay for pap smear test			
Not willing to pay		462	30.8
300–500 SAR		935	62.4
>500 SAR		101	6.70
2	Received HPV vaccine		
Yes ^1^		3	0.20
No		1253	83.6
I do not know		242	16.2

HPV: human papilloma virus; ^1^ correct response.

**Table 3:** Predictors of awareness related to cervical cancer.

	B	SE	*p*-Value	95% Confidence Interval	R^2^
Age group	0.43	0.16	0.007 *	0.12 to 0.74	0.01
Marital status	0.55	0.16	0.001 *	0.24 to 0.86	0.01
Education level	0.26	0.17	0.122	−0.07 to 0.59	0.00
Employment status	−1.21	0.10	<0.001 *	−1.41 to −1.01	0.08
Family income per month	0.60	0.09	<0.001 *	0.44 to 0.77	0.03
Region of residency	−0.55	0.20	0.006 *	−0.93 to −0.16	0.01

* α = 0.01.

**Table 4:** Predictors of performing the pap smear test.

	OR	95% Confidence Interval	*p*-Value
Age			
18–25 years >25 years	Reference category		
	16.8	9.66 to 29.3	<0.001 *
Marital status			
Single Married/divorced or widowed	Reference category		
	69.4	22.1 to 218	<0.001 *
Education level			
≤High school/diploma Bachelor’s/postgraduate degree	Reference category		
	0.12	0.87 to 1.71	<0.001 *
Employment status			
Employee or student in medical field Employee or student in nonmedical field Not employee or student	Reference category		
	2.04	1.21 to 3.44	0.007 *
	2.93	1.75 to 4.92	<0.001 *
Family income per month			
<10,000 SAR 10,000 SAR >10,000 SAR	Reference category		
	0.69	0.41 to 1.18	0.176
	1.40	0.99 to 1.96	0.054
Region of residency			
Al Madinah Al Munawwarah city Other cites	Reference category		
	1.60	1.13 to 2.27	<0.001 *

* α = 0.01.

## Data Availability

The data presented in this study are available on request from the corresponding author. The data are not publicly available.
